# MPRO3 Regulates Macrophage Polarization and Osteoclastogenesis *In Vitro* and Attenuates Ligature-Induced Alveolar Bone Loss in Periodontitis

**DOI:** 10.4014/jmb.2604.04011

**Published:** 2026-08-14

**Authors:** Hyun-Joo Park, Tae Sung Kim, Mi-Kyoung Kim, Yeon Kim, Soon Chul Heo, Dong Ki Hong, Soo Dong Park, Jae Jung Shim, Jae Hwan Lee, Hyung Joon Kim, Soo-Kyung Bae, Moon-Kyoung Bae

**Affiliations:** 1 Department of Oral Physiology, School of Dentistry, Pusan National University, Yangsan, 50612, Republic of Korea; 2 Dental and Life Science Institute, Pusan National University, Yangsan, 50612, Republic of Korea; 3 Department of Oral Microbiology, School of Dentistry, Pusan National University, Yangsan, 50612, Republic of Korea; 4 R&BD Center, hy Co., Ltd., Yongin-si 17086, Republic of Korea; 5 Department of Dental Pharmacology, School of Dentistry, Pusan National University, Yangsan, 50612, Republic of Korea

**Keywords:** Macrophage polarization, Osteoclastogenesis, Periodontal disease

## Abstract

Periodontal disease is a chronic inflammatory disorder characterized by the destruction of periodontal tissues. It is caused by microbial biofilm deposition and altered host immune responses. Prebiotics, probiotics, and postbiotics have recently gained attention as complementary therapeutic strategies against periodontal disease. Here, we investigated whether MPRO3, a formulation containing heat-killed *Bifidobacterium animalis* subsp. *lactis*, *Lactiplantibacillus plantarum*, and *Lacticaseibacillus paracasei*, could mitigate periodontal inflammation and bone resorption. Treatment of THP-1 cells with MPRO3 attenuated *Porphyromonas gingivalis* lipopolysaccharide-induced M1 polarization, while enhancing M2 polarization instead. In addition, MPRO3 suppressed osteoclast differentiation of bone marrow-derived macrophages and regulated the expression of osteoclastogenesis-related factors. In a mouse model of ligature-induced periodontitis, MPRO3 significantly attenuated alveolar bone loss, accompanied by decreased TRAP-positive osteoclasts, reduced RANKL expression, and lower systemic pro-inflammatory cytokine levels. Collectively, these findings indicate that MPRO3 regulates macrophage polarization and osteoclastogenesis, highlighting its potential as an adjunctive intervention in periodontal disease management.

## Introduction

Periodontitis is a chronic inflammatory condition of the periodontium, characterized by progressive destruction of the connective tissue and alveolar bone that anchor the teeth [1]. The ensuing inflammatory and immune cascades then progressively degrade tooth-supporting structures [2]. In addition to local tissue damage, this chronic inflammatory state is increasingly linked to systemic diseases, including diabetes mellitus, cardiovascular disease, and rheumatoid arthritis [3].

Macrophages play a pivotal role in the pathogenesis of various inflammatory diseases, including periodontitis, by polarizing into pro-inflammatory M1 and anti-inflammatory M2 phenotypes [4]. In chronic lesions, the predominance of M1 over M2 responses sustains inflammation and promotes progression of periodontal disease [5]. Osteoclasts, multinucleated cells formed by the fusion of monocytes and macrophage precursors, mediate bone resorption and maintain skeletal homeostasis [6]. Aberrant osteoclastogenesis accelerates alveolar bone destruction and ultimately compromises tooth support in periodontitis [7, 8].

Recent evidence indicates that probiotics and postbiotics, including heat-killed probiotic preparations, exert beneficial effects irrespective of bacterial viability, targeting growth and biofilm formation by pathogenic bacteria, microbial adhesion, and periodontal inflammation caused by host immune responses [9-11]. Postbiotics have been proposed as non-viable alternatives to probiotics because they may offer practical advantages, including improved safety, enhanced stability, and greater ease of storage and formulation standardization [12, 13]. In addition, preclinical and clinical studies have demonstrated the potential periodontal benefits of heat-killed bacterial preparations [14, 15].

This study examined the effects of MPRO3, a postbiotic preparation composed of heat-killed *B. animalis* subsp. *lactis*, *L. plantarum*, and *L. paracasei*, on macrophage M1/M2 polarization and osteoclast differentiation *in vitro*, as well as its potential effects in an *in vivo* model of experimental periodontitis.

## Materials and Methods

### Reagents and Antibodies

Recombinant receptor activator of nuclear factor κB ligand (RANKL) and macrophage colony-stimulating factor (M-CSF) were sourced from PROSPEC (East USA) and PeproTech (USA), respectively. Tartrate-resistant acid phosphatase (TRAP) activity was detected using a commercially available staining kit from Sigma-Aldrich (USA). Flow cytometric analyses employed PerCP/Cyanine5.5-labeled antibodies against CD80 and CD86 (BioLegend, USA). Antibodies against total and phosphorylated forms of ERK, p38 MAPK, JNK, AKT, STAT1, STAT6, and phospho-p65 were purchased from Cell Signaling Technology (USA). Antibodies recognizing total p65, nuclear factor of activated T cell cytoplasmic 1 (NFATc1), and β-actin were obtained from Santa Cruz Biotechnology (USA) and Bioworld Technology (USA), respectively. Horseradish peroxidase–conjugated secondary antibodies (goat anti-rabbit and anti-mouse IgG) were obtained from Thermo Fisher Scientific (USA).

### Postbiotic Preparation

The commercial probiotic/synbiotic MPRO3 formulation previously described consists of *L. plantarum* HY7712, *L. paracasei* HY2782, and *B. animalis* subsp. *lactis* HY8002 [16]. In the present study, these strains were prepared as a non-viable postbiotic formulation. Specifically, heat-killed *L. plantarum* HY7712, heat-killed *L. paracasei* HY2782, and heat-killed *B. animalis* subsp. *lactis* HY8002 were mixed at a weight ratio of 1:1:2, respectively [17]. Each strain was individually cultured in a MRS medium (De Man, Rogosa and Sharpe medium, MilliporeSigma) at 37 ± 4°C for 24 ± 6 h, followed by heat inactivation at 121 ± 6°C for 20 ± 10 min. An excipient was added before drying the mixture into a powder form. Before use, the preparations were reconstituted in phosphate-buffered saline (PBS).

### Macrophage Polarization

The human monocytic THP-1 cell line was obtained from the Korea Cell Line Bank (Republic of Korea) and maintained in RPMI 1640 medium (Gibco, USA) supplemented with 10% fetal bovine serum (Gibco), 1% penicillin–streptomycin (Gibco), and 5 μg/mL Plasmocin^®^ (Invitrogen, USA). Cells were cultured at 37°C in a humidified incubator containing 5% CO_2_. THP-1 cells were treated with 150 nM phorbol 12-myristate 13-acetate (PMA; Sigma-Aldrich) for 24 h to induce differentiation into resting (M0) macrophages. To induce M1 polarization, M0 macrophages were stimulated with 1 μg/mL *Porphyromonas gingivalis* lipopolysaccharide (*P. gingivalis* LPS; InvivoGen, USA) and 20 ng/mL interferon-γ (IFN-γ; R&D Systems, USA), after which cells were harvested at the indicated time points. For M2 polarization, M0 macrophages were treated with 20 ng/mL each of interleukin (IL)-4 and interleukin-13 (IL-13) (R&D Systems).

### *In Vitro* Osteoclast Differentiation

Bone marrow-derived macrophages (BMMs) were isolated from the femurs and tibiae of 5-week-old ICR mice and used as osteoclast precursor cells. Briefly, bone marrow was flushed from long bones using α-minimum essential medium (α-MEM) and erythrocytes were eliminated from the collected cell suspensions by lysis. The remaining cells were seeded into culture dishes and maintained overnight in α-MEM containing 10% fetal bovine serum and 1% antibiotics. The following day, non-adherent cells were collected and replated on medium supplemented with 30 ng/mL M-CSF. After 3 days of culture, adherent cells were gently detached using a cell scraper and designated as osteoclast precursors. To induce osteoclast differentiation, these cells were cultured for 5 days in osteoclastogenic medium consisting of α-MEM supplemented with 30 ng/mL M-CSF and 100 ng/mL RANKL. Culture medium was replaced every 2–3 days. Osteoclast formation was evaluated by TRAP staining at the end of the differentiation period.

### Bone Resorption Assay

Osteoclastic bone-resorbing activity was evaluated using calcium phosphate-coated bone resorption assay plates (Cosmo Bio Co. Ltd., Japan). BMMs were seeded onto coated plates and cultured in osteoclastogenic medium in the presence or absence of MPRO3 for 5 days to allow osteoclast formation. Upon completion of differentiation, the cells were removed by treatment with 5% sodium hypochlorite. The plates were then rinsed thoroughly with distilled water and air-dried at room temperature. Resorption lacunae were visualized under a (OPTINITY, Korea Lab Tech. Republic of Korea) and the resorbed areas were quantified using ImageJ software (NIH, USA).

### Reverse Transcription-Quantitative PCR (RT-qPCR)

Total RNA was extracted using RiboEx reagent (GeneAll, Republic of Korea) according to the manufacturer’s instructions. Complementary DNA was synthesized from isolated RNA using a reverse transcription kit (Promega, USA). Quantitative real-time PCR was conducted using SYBR Green-based premix (Enzynomix, Republic of Korea) in a real-time PCR system (Applied Biosystems, USA) under the following thermal cycling conditions: an initial denaturation step at 95°C for 10 min, followed by 40 cycles of denaturation at 95°C for 15 s, annealing at 60°C for 60 s, and extension at 72°C for 7 s. Gene expression was quantified using the following gene-specific primers: mouse β-actin, 5′-TCTGGCACCACACCTTCTAC-3′ and 5′-TCTGGCACCACACCTTCTAC-3′ and 5′-TACGACCAGAGGCATACAGG-3′; mouse TRAP, 5′-CGACCATTGTTA GCCACATACG-3′ and 5′-TCGTCCTGAAGATACGGTT-3′; mouse cathepsin K (CTK), 5′-ATATGTGGGCCACCATGAAAGTT-3′ and 5′-TCGTT CCCCACAGGAATCTCT-3′; mouse dendritic cell-specific transmembrane protein (DC-STAMP), 5′-GGGTGCTGTTTGCCGCTG-3′ and 5′-CGACTCCTTGGGTTCCTTGCT-3′; mouse RANKL, 5′-AGGCTGGGCCAAGATCTCTA-3′ and 5′-GTCTGTAGGTACGCTTCCCG-3′; mouse osteoprotegerin (OPG), 5′-GCAGCCCAGTGTGCAAGGAA-3′ and 5′-TTCGATCTCCAGGTAACGCCC-3′; human β-actin, 5′-ACTCTTCCAG CCTTCCTTCC-3′ and 5′-TGTTGGCGTACAGGTCTTTG-3′; human CD80, 5′-CGGAGGCAGGGAACATCACC-3′ and 5′-TGACCAC AGGACAGCGTTGC-3′; human CD86, 5′-TCGTGATGGCCTTCCTGCTC-3′ and 5′-TGCAAATTGGCAAGGCAGGT-3′; human CD163, 5′-TGGCATGCAAGCAACTGGGA-3′ and 5′-GCAGGTTCATGTCCCTGGCA-3′; human CD206, 5′-GCCGTATGCCGGTCACTGTT-3′ and 5′-AGGTCACCGCCTTCCTTCCT-3′; human IL-6, 5′-CTAGAGTACCTCCAGAACAGATTTGA-3′ and 5′-TCAGCAGGCTGGCATTT-3′; human IL-1β, 5′-GACCTGGACCTCTGCCCTCT-3′ and 5′-CTGCCTGAAGCCCTTGCTGT-3′; human tumor necrosis factor (TNF)-α, 5′-CAGGCGGTGCTTGTTCCTCA-3′ and 5′-AGTGCAGCAGGCAGAAGAGC-3′; and human IL-10, 5′-TTAAGGGTTACCTGGGTTGC-3′ and 5′-TTCA CGTGCTCCTTGATGTC-3′.

### Western Immunoblot Assay

Equal amounts of total protein (approximately 30 μg per lane) were resolved on 10% bis-acrylamide gels by SDS-PAGE. Proteins were subsequently transferred onto nitrocellulose membranes (Amersham Pharmacia Biotech, Sweden). Membranes were blocked with 5% skim milk for 1 h at room temperature with gentle agitation and then incubated with the appropriate primary antibodies for 16 h at 4°C. After incubation, the membranes were washed three times with PBS for 10 min each and probed with horseradish peroxidase–conjugated goat anti-rabbit IgG or goat anti-mouse IgG secondary antibodies for 1 h at room temperature. Immunoreactive bands were detected using an enhanced chemiluminescence detection reagent (Amersham Pharmacia Biotech) and visualized with an Azure 300 imaging system (Azure Biosystems, USA).

### Flow Cytometry

M0 macrophages were polarized toward M1 or M2 phenotypes in the presence or absence of MPRO3. Following 48 h of polarization, cells were harvested and stained for 1 h at 4°C with either PerCP/Cyanine5.5-conjugated anti-CD86 antibody (2 μg/mL) for M1 macrophages or PerCP/Cyanine5.5-conjugated anti-CD206 antibody (2 μg/mL) for M2 macrophages. Finally, cells were washed twice with PBS and analyzed using a flow cytometer (BD Biosciences, USA).

### Enzyme-Linked Immunosorbent Assay (ELISA)

Cytokines secreted into conditioned medium were quantified using a commercially available human pro-inflammatory cytokine multiplex ELISA kit (Arigo Biolaboratories, Taiwan) for IL-6, IL-1β, and TNF-α; and an individual ELISA kit (ABclonal, USA) for IL-10. Optical density was measured at 450 nm using a microplate reader from BIOAND (Republic of Korea) for multiplex assays or from Dynex Technologies (USA) for IL-10. Cytokine concentrations were calculated by comparing with a standard curves generated following assay instructions.

### Ligature-Induced Periodontitis in Mice

All animal procedures were conducted in compliance with the established guidelines for laboratory animal care and were approved by the Institutional Animal Care and Use Committee of Pusan National University (approval no. PNU-2024-0401). Six-week-old male C57BL/6J wild-type mice (22–25 g) were obtained from Orient Co., Ltd. (Republic of Korea) and housed under standard laboratory conditions. Experimental periodontitis was induced using a ligature model, as previously described [18]. Briefly, a 5–0 silk ligature (AILEE Inc., Republic of Korea) was placed around the left maxillary second molar and secured with a surgical knot. Mice were randomly assigned to experimental groups (n = 7 per group). One day after ligature placement, corresponding to the early inflammatory phase of ligature-induced periodontitis, the mice received MPRO3 at 1 × 10^9^ cells by daily oral gavage for 14 consecutive days. The animals were sacrificed on day 14, and maxillary tissues were harvested for subsequent analyses. The excised maxillae were fixed in 4% paraformaldehyde and scanned using a micro-computed tomography system (MicroCT.SMX-90 CT; Shimadzu Corp., Japan). Scans were acquired over a 360° rotation at 90 kV and 100 mA, and three-dimensional reconstructions were generated using inspeXio SMX-90CT software (Shimadzu Corp.). Sagittal images of maxillary second molars were obtained using the apices of the mesiobuccal and distobuccal roots as anatomical landmarks. Alveolar bone loss was quantified by measuring the distance from the palatal and mesiobuccal cemento–enamel junction (CEJ) to the alveolar bone crest (ABC). Maxillary tissues were embedded in cryomolds with optimal cutting temperature (OCT) compound, frozen, and sectioned at a thickness of 5 μm using a cryostat (MICROM HM525, Thermo Scientific). For TRAP staining, tissue sections were stained using an acid phosphatase kit (Sigma-Aldrich). Total RNA was extracted from mouse gingival tissues using an RNeasy kit (Qiagen GmbH, Germany) following the manufacturer’s protocol.

### Statistical Analysis

All quantitative data are presented as the mean ± standard deviation of at least three independent experiments. Statistical comparisons were performed using one-way analysis of variance, followed by Tukey’s honest significant difference post hoc test or Student’s *t*-test, as appropriate. All analyses were conducted using IBM SPSS software version 27 (IBM Corp., USA).

## Results

### MPRO3 Modulates the Expression of M1 and M2 Markers during Macrophage Polarization

**Fig. 1:**
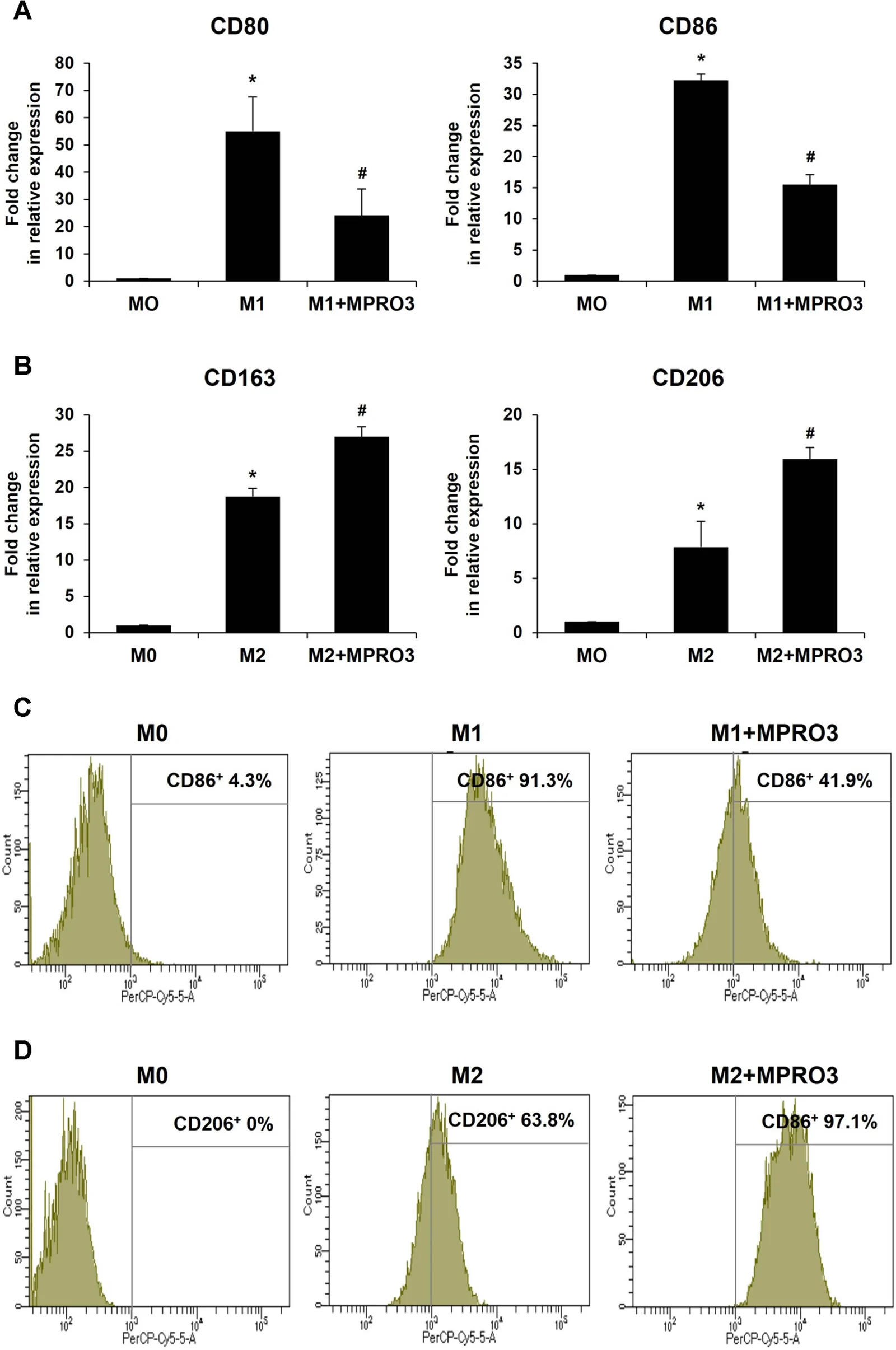
Effect of MPRO3 on macrophage polarization toward M1 and M2 phenotypes. (**A, B**) THP-1-derived macrophages were subjected to M1-polarizing stimuli (1 μg/mL *P. gingivalis* LPS plus 20 ng/mL IFN-γ) or M2-polarizing stimuli (20 ng/mL IL-4 and 20 ng/mL IL-13) in the presence or absence of MPRO3 for 48 h. Transcript levels of the M1 markers CD80 and CD86 and the M2 markers CD163 and CD206 were determined by RT-qPCR, normalized to β-actin, and expressed relative to control values. (**C, D**) Cell-surface expression of CD86 in M1 macrophages and CD206 in M2 macrophages was evaluated by flow cytometry. Data are shown as mean ± standard deviation. *P < 0.05 *versus* M0; ^#^*P* < 0.05 versus polarized control.

To clarify the role of MPRO3 in macrophage polarization, we assessed its effect on the viability of THP-1-derived M0 macrophages. Human THP-1 monocytes were differentiated into resting macrophages (M0) using PMA and subsequently exposed to MPRO3 at 1 to 200 MOIs. Cell viability was assessed over a 3-day period using an MTT assay. MPRO3 treatment did not induce significant cytotoxicity at an MOI of 1; whereas exposure to higher MOIs (≥10) resulted in a time-dependent decline in cell viability (Fig. S1). Therefore, an MOI of 1 was used for all subsequent experiments. Next, we investigated whether MPRO3 influenced macrophage polarization toward the M1 or M2 state. In the presence of MPRO3, M0 macrophages were polarized toward M1 macrophages, using *P. gingivalis* LPS combined with IFN-γ, or toward M2 macrophages, using IL-4 and IL-13. As expected, M1 polarization led to significantly higher mRNA expression of the M1 markers CD80 and CD86; however, MPRO3 treatment markedly attenuated this trend (Fig. 1A). Conversely, expression of the M2 markers CD163 and CD206 was substantially elevated under M2-polarizing conditions, and was further enhanced by MPRO3 treatment (Fig. 1B). These transcriptional changes were supported by flow cytometry data, which showed significantly reduced surface CD86 expression in M1 macrophages (Fig. 1C) and increased CD206 expression in M2 macrophages (Fig. 1D) following MPRO3 supplementation.

### MPRO3 Differentially Regulates Cytokine Production and associated Signaling Pathways during Macrophage Polarization

**Fig. 2:**
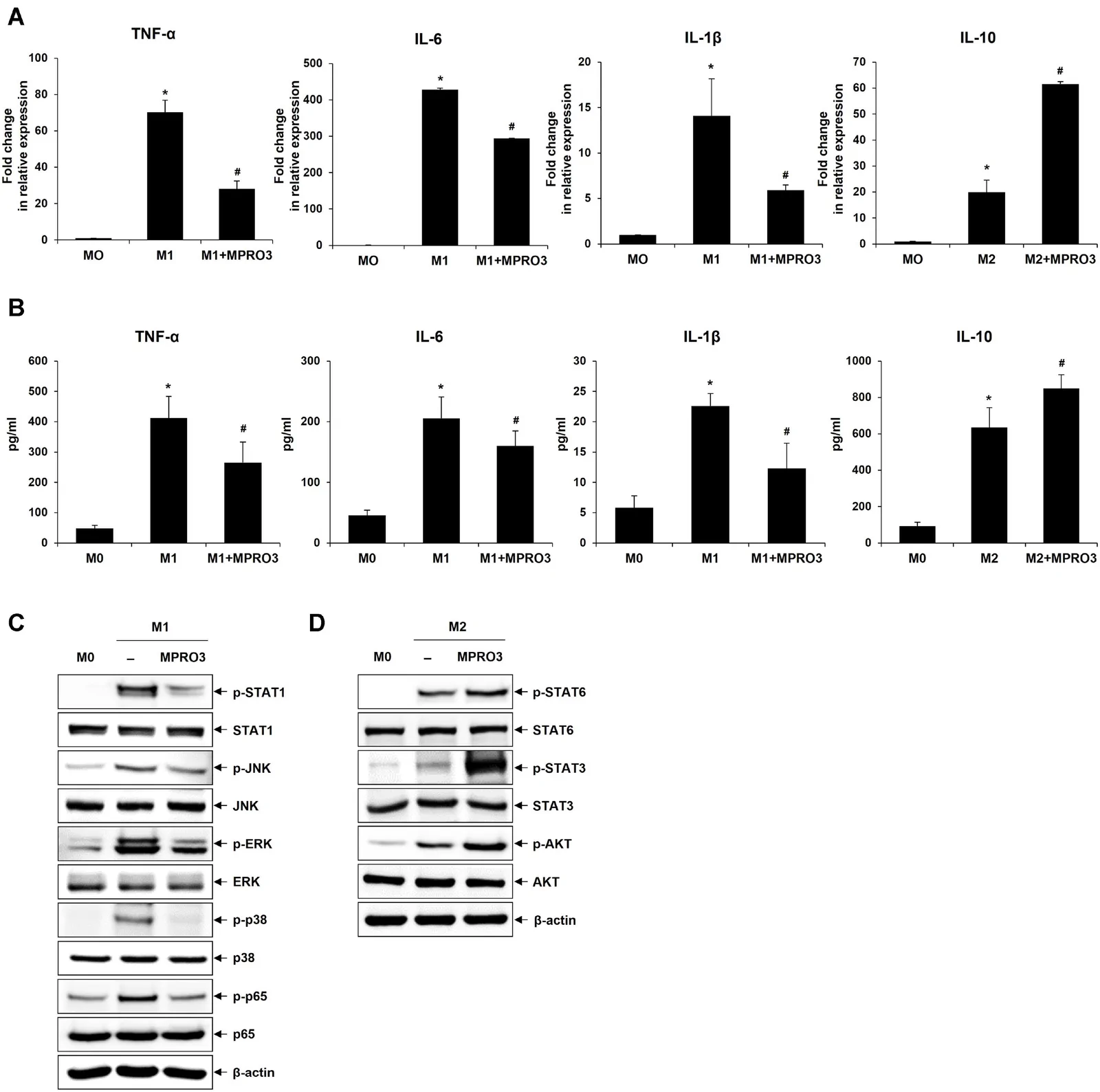
Effect of MPRO3 on inflammatory cytokine production and associated signaling pathways during M1 and M2 macrophage polarization. THP-1-derived macrophages were cultured under M1- or M2-polarizing conditions with or without MPRO3 for 48 h. (**A**) Relative mRNA expression of TNF-α, IL-6, IL-1β, and IL-10 was analyzed by RT-qPCR after 48 h of polarization and normalized to β-actin. (**B**) Secretion of TNF-α, IL-6, IL-1β, and IL-10 into culture supernatant was quantified by ELISA. For signaling analyses, macrophages were polarized for 24 h and MPRO3 was added during the final hour. (**C, D**) Total and phosphorylated forms of STAT1, JNK, ERK, p38, p65, STAT6, and AKT were examined by western blotting, using β-actin as loading control. Quantitative data are presented as mean ± standard deviation. *P < 0.05 *versus* M0; ^#^*P* < 0.05 versus polarized control.

Polarized macrophages exhibit distinct cytokine production patterns that reflect their functional heterogeneity, with M1 macrophages predominantly expressing pro-inflammatory cytokines and M2 macrophages expressing anti-inflammatory cytokines [19]. Accordingly, we examined whether MPRO3 regulated cytokine expression during macrophage polarization. As illustrated in Fig. 2A, mRNA levels of the pro-inflammatory cytokines TNF-α, IL-6, and IL-1β were markedly increased in M1-polarized macrophages, but declined significantly upon MPRO3 treatment. In contrast, MPRO3 exposure resulted in a pronounced increase in IL-10 expression in M2 macrophages. Consistent with the observed mRNA expression patterns, ELISA demonstrated corresponding alterations in the secretion of TNF-α, IL-6, IL-1β, and IL-10 (Fig. 2B). STAT1 activation and MAPK pathways, such as JNK, ERK, and p38, play a central role in regulating pro-inflammatory signaling and cytokine expression in macrophages [20, 21]. Therefore, we tested whether MPRO3 modulated STAT1 and MAPK, and NF-κB signaling pathways in M1-polarized macrophages. As shown in Fig. 2C, M1 polarization was associated with increased phosphorylation of STAT1, JNK, ERK, p38, and p65, all of which were significantly attenuated by MPRO3 treatment. In contrast, M2 macrophage polarization is associated with the activation of the STAT3, STAT6, and AKT signaling pathways linked to anti-inflammatory responses [22, 23]. MPRO3 treatment enhanced the phosphorylation of these signaling molecules (Fig. 2D).

### MPRO3 Inhibits RANKL-Induced Osteoclast Differentiation and Bone Resorption *In Vitro*

To evaluate the effect of MPRO3 on cell viability of BMMs, the impact of various MOIs was assessed using the MTT assay over a 3-day period. Because MPRO3 exhibited no significant cytotoxicity at an MOI of 1, this concentration was used in all subsequent experiments (Fig. S2).

**Fig. 3:**
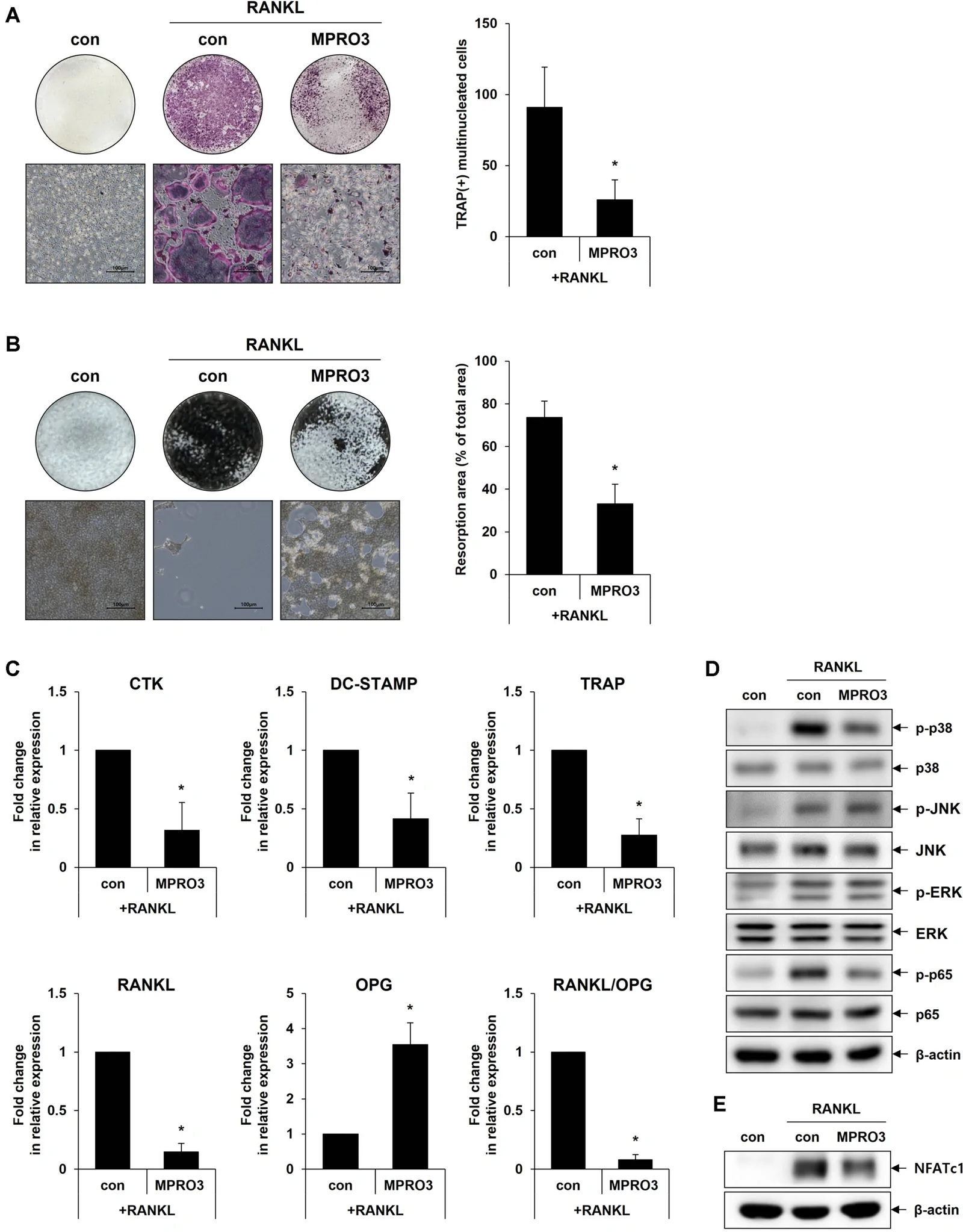
Effect of MPRO3 on ostoclstogenesis in BMMs. (**A**) BMMs were cultured with differentiation medium in the presence or absence of MPRO3 for 5 days. Following the differentiation period, osteoclastogenesis was quantified by counting TRAP-positive multinucleated cells under a light microscope. (**B**) BMMs were seeded on bone resorption assay plates and stimulated with RANKL with or without MPRO3 for 5 days. The resorption areas were visualized, and the relative resorbed surface area was measured using ImageJ software. (**C**) The mRNA expression of key osteoclastogenic marker genes was analyzed by RT-qPCR after 5 days of differentiation. (**D**) BMMs were incubated with MPRO3 and RANKL for 10 min. The phosphorylation of p38, ERK, JNK, and p65 was analyzed by western blotting. (**E**) NFATc1 expression was determined by Western blotting in BMMs incubated with MPRO3 and RANKL. All data are presented as mean ± standard deviation; *P < 0.05 *versus* RANKL-only treated group.

To quantify osteoclast differentiation and functional activity, BMMs were cultured with RANKL in the presence or absence of MPRO3 for 5 days, followed by TRAP staining and a bone resorption assay. As shown in Fig. 3A, RANKL-induced osteoclastogenesis was significantly inhibited in MPRO3-treated cells, as indicated by fewer multinucleated TRAP-positive cells. Consistent with its negative effect on osteoclastogenesis, MPRO3 significantly also reduced the resorbed area (Fig. 3B). Hence, we determined the expression of osteoclast-specific marker genes. MPRO3 considerably downregulated the mRNA levels of TRAP, DC-STAMP, and CTK compared to those in the RANKL-only group. In addition, MPRO3 decreased RANKL expression while increasing OPG expression, resulting in a significant reduction in the RANKL/OPG ratio (Fig. 3C). To investigate the signaling mechanisms underlying osteoclast differentiation, we assessed the activation of the MAPK pathways (p38, ERK, and JNK) and NF-κB, which are essential for osteoclast differentiation and survival [24-26]. Western blot analysis demonstrated that MPRO3 significantly suppressed RANKL-induced phosphorylation of p38MAPK, but had no effect on phosphorylation of ERK and JNK. Furthermore, MPRO3 treatment effectively attenuated the phosphorylation of p65, a functional subunit of the NF-κB pathway (Fig. 3D). Because NFATc1 is a master regulator of osteoclastogenesis regulated by these upstream signals, we further evaluated its protein expression [27]. As shown in Fig. 3E, MPRO3 effectively suppressed the protein expression of NFATc1 induced by RANKL.

### MPRO3 Attenuates Periodontal Inflammation and Alveolar Bone Loss in a Ligature-Induced Periodontitis Model

**Fig. 4:**
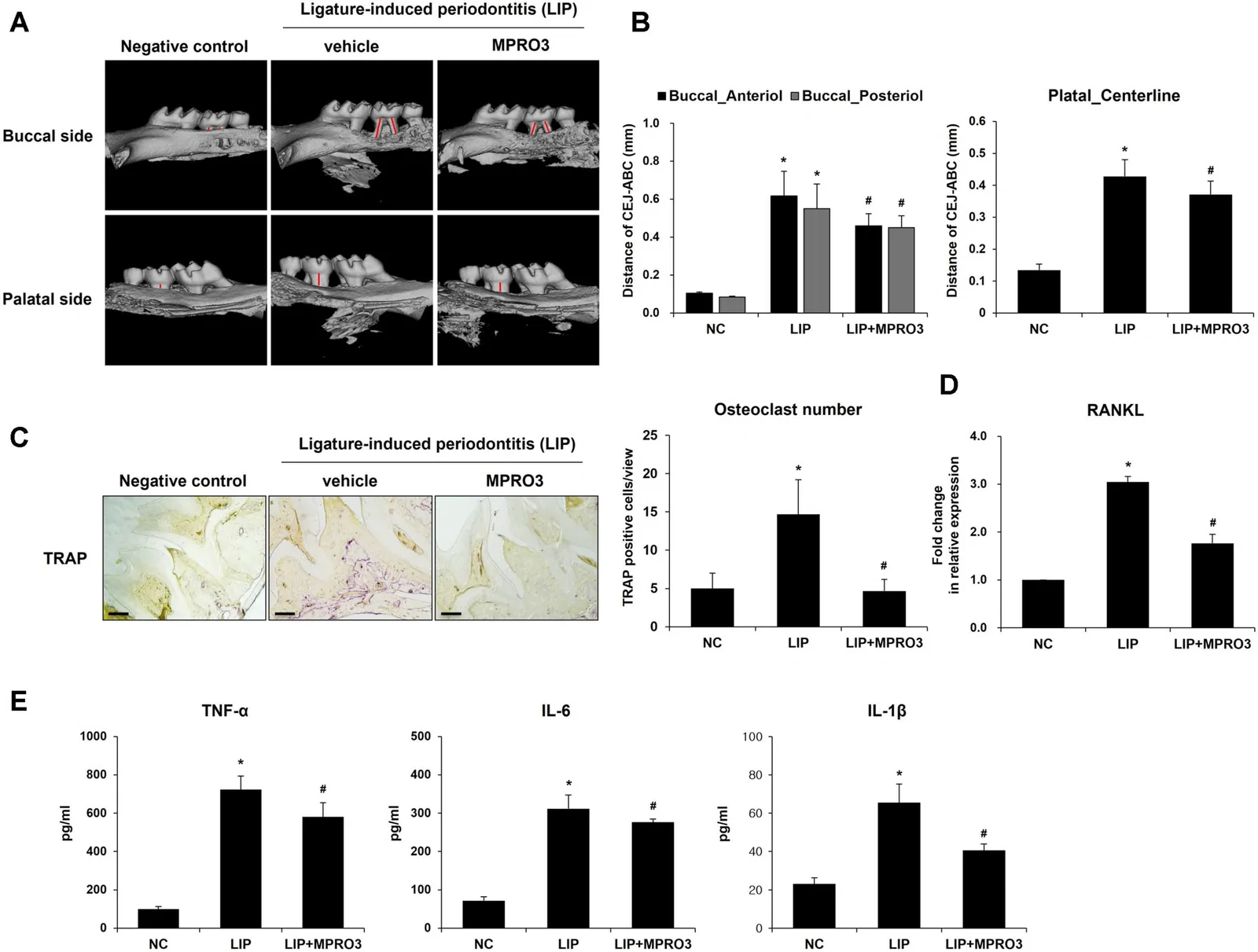
Effect of MPRO3 on alveolar bone loss and inflammatory response in a ligature-induced mouse model. Ligatures were placed around the left maxillary second molars, followed by daily oral administration of MPRO3 (1 × 10^9^ cells) for 14 days. Maxillary samples were collected at the end of the experimental period. (**A**) Representative micro-computed tomography images showing alveolar bone loss around the ligated maxillary second molars. (**B**) Alveolar bone loss was quantitatively evaluated by measuring the CEJ–ABC distance at multiple buccal and palatal sites using three-dimensional reconstructions. (**C**) Representative TRAP staining of alveolar bone sections (left) and quantitative analysis of TRAP-positive osteoclasts at the alveolar crest (right). Black bar = 100 μm. (**D**) Relative expression of RANKL mRNA in gingival tissues were analyzed by RT-qPCR and normalized to β-actin. (**E**) Circulating TNF-α, IL-6, and IL-1β were determined by ELISA. All quantitative data are presented as mean ± standard deviation. *P < 0.05 *versus* control; ^#^*P* < 0.05 *versus* ligature-treated group.

A ligature-induced periodontitis model was used to evaluate the in vivo effect of MPRO3 on periodontal inflammation and alveolar bone loss [18]. Following ligature placement around the right maxillary second molar, the mice were orally administered MPRO3 daily for 14 days. Micro-computed tomography demonstrated that ligature placement resulted in substantial alveolar bone loss, with pronounced bone resorption in the vehicle-treated group. In contrast, MPRO3 treatment significantly preserved alveolar bone height (Fig. 4A). Quantitative assessment of periodontal destruction using the CEJ–ABC distance further confirmed these findings, showing a marked increase in the vehicle group, which was significantly reduced following MPRO3 treatment (Fig. 4B). In parallel, histological analysis using TRAP staining demonstrated elevated osteoclast formation at the alveolar crest two weeks after ligation, which was substantially suppressed by MPRO3 (Fig. 4C). Immunohistochemical analysis further showed that expression of inducible nitric oxide synthase (iNOS), an M1 macrophage-associated marker [28], in gingival tissues was increased following ligature placement and attenuated by MPRO3 treatment (Fig. S3). RT-qPCR analysis of gingival tissues revealed that ligature-induced periodontitis markedly elevated the mRNA expression of RANKL, a key regulator of osteoclastogenesis. This induction was significantly suppressed in mice treated with MPRO3 (Fig. 4D). Systemic inflammatory responses in a ligature-induced periodontitis mouse were evaluated by measuring circulating levels of pro-inflammatory cytokines with ELISA. Serum concentrations of TNF-α, IL-6, and IL-1β were substantially increased in ligature-treated mice, but were significantly reduced following MPRO3 treatment (Fig. 4E).

## Discussion

Dysbiosis of the oral microbiome contributes to the initiation and progression of periodontitis, largely through disruption of host–microbe homeostasis [29]. Recently, microbiome-targeting strategies, including probiotics, synbiotics, and postbiotics, have emerged as promising adjunctive agents for periodontal therapy [30, 31]. They can exert beneficial effects not only by modulating the host immune response, but also by directly inhibiting growth, colonization, and biofilm formation by periodontal pathogens [32, 33]. Postbiotic preparations derived from *Lactobacillus* species have been reported to inhibit the establishment of pathogenic biofilms [34]. In particular, heat-killed *Lactobacillus reuteri* and its cell-free culture supernatant were shown to exert effects comparable to those of viable probiotics during interaction with *P. gingivalis*, supporting the functional relevance of non-viable bacterial preparations [35]. Furthermore, both viable and heat-killed *L. reuteri* reduced alveolar bone loss in a rat model of experimentally induced periodontitis, providing *in vivo* support for the potential periodontal benefits of heat-killed bacterial preparations [15]. In the present study, we demonstrated that treatment with MPRO3 regulated M1/M2 macrophage polarization and suppressed osteoclast differentiation *in vitro*, indicating that MPRO3 affected the inflammatory and bone resorptive processes associated with periodontal disease. Although the present study did not directly evaluate antimicrobial or antibiofilm activity, the immunomodulatory and anti-osteoclastogenic effects observed here support the potential of MPRO3 as a modulatory agent. Further studies should determine whether MPRO3 possesses antimicrobial properties and inhibits pathogenic biofilm formation, which would further expand its applications in periodontal disease management.

Periodontal disease is influenced not only by dysbiosis of the oral microbiome, but also by alterations of the gut microbiota [36, 37]. Previous studies have suggested that probiotics and postbiotics may influence oral microbial balance and periodontal health through modulation of oral biofilms and host immune responses [30, 38]. In addition, alterations in gut microbial composition may contribute to systemic inflammatory and immune responses that could influence periodontal inflammation [39, 40]. Although MPRO3 showed beneficial effects on periodontal inflammation in the present study, direct analyses of oral or gut microbiota were not performed. Therefore, the potential involvement of microbiome-associated mechanisms remains to be further elucidated. Additional studies investigating the effects of MPRO3 on oral microbial composition are currently underway.

Although RANKL stimulation activates multiple MAPK signaling pathways, including ERK, JNK, and p38 through TRAF6-dependent signaling [41], our results demonstrated that MPRO3 selectively inhibited p38 phosphorylation, whereas ERK and JNK activation were not significantly affected. Previous studies have shown that p38 MAPK plays a critical role in osteoclast differentiation by regulating key osteoclastogenic transcription factors, including c-Fos and NFATc1, which are essential for osteoclast formation and function [42, 43]. Notably, MPRO3 also reduced the RANKL-induced expression of NFATc1, a key transcription factor required for osteoclast differentiation in this study. Therefore, the selective suppression of p38 signaling by MPRO3 may be sufficient to attenuate RANKL-induced osteoclastogenesis. In addition, MPRO3 also inhibited NF-κB activation, suggesting that its anti-osteoclastogenic effect may involve selective modulation of specific downstream signaling pathways rather than broad inhibition of the entire RANK/TRAF6 signaling cascade. Nevertheless, the precise molecular mechanism underlying the selective inhibition of p38 by MPRO3 remains to be further elucidated.

A limitation of the present study in ligature-induced periodontitis model is that MPRO3 administration was initiated 1 day after ligature placement, which may partly reflect intervention during the early phase of disease progression rather than treatment of fully established chronic periodontitis. Nevertheless, previous studies have demonstrated that inflammatory infiltrates, pro-inflammatory cytokine expression, and initial bone resorption can already be detected within 1–3 days after ligature placement [44]. Therefore, MPRO3 administration in the present study may still reflect modulation of ongoing inflammatory and destructive processes. Future studies employing delayed administration after establishment of periodontitis will be necessary to further evaluate the effects of MPRO3 in established disease.

Taken together, findings from the present study demonstrate that MPRO3 regulates macrophage polarization, suppresses osteoclast differentiation in vitro, and attenuates periodontal inflammation and bone loss in a ligature-induced periodontitis model. These findings suggest the potential applicability of MPRO3 in periodontal management. However, further studies comparing MPRO3 with clinically established agents, as well as combination studies evaluating potential synergistic effects, will be necessary to determine its translational and clinical applicability. Ultimately, additional clinical studies involving human subjects will be required to validate its therapeutic potential.

## Supplemental Materials

Supplementary data for this paper are available on-line only at http://jmb.or.kr.
