# Supplementary material for: MPRO3 Regulates Macrophage Polarization and Osteoclastogenesis In Vitro and Attenuates Ligature-Induced Alveolar Bone Loss in Periodontitis
Source: J Microbiol Biotechnol. 2026 Aug 14;36:e2604011. doi: 10.4014/jmb.2604.04011 (PMC13519251; doi:10.4014/jmb.2604.04011)
Supplement: Supplementary file 1 [file jmb-36-e2604011-supple.pdf]

J. Microbiol. Biotechnol. 2026. 36: e2604011

https://doi.org/10.4014/jmb.2604.04011

# MPRO3 regulates macrophage polarization and osteoclastogenesis *in vitro* and attenuates ligature-induced alveolar bone loss in periodontitis

Hyun-Joo Park [1,2†], Tae Sung Kim[3†], Mi-Kyoung Kim [1], Yeon Kim[1,2], Soon Chul Heo[1], Dong Ki Hong[4], Soo Dong Park[4], Jae Jung Shim[4], Jae Hwan Lee[4], Hyung Joon Kim [1,2], Soo-Kyung Bae[2,5], Moon-Kyoung Bae[1,2*]

[1] *Department of Oral Physiology, School of Dentistry, Pusan National University, Yangsan, 50612, Republic of Korea*

[2] *Dental and Life Science Institute, Pusan National University, Yangsan, 50612, Republic of Korea*

[3] *Department of Oral Microbiology, School of Dentistry, Pusan National University, Yangsan, 50612, Republic of Korea.*

[4] *R&BD Center, hy Co., Ltd., Yongin-si 17086, Republic of Korea.*

[5] *Department of Dental Pharmacology, School of Dentistry, Pusan National University, Yangsan, 50612, Republic of Korea.*

[†] These authors contributed equally to this work.

# Supplementary Materials and Methods

## MTT assay

Cell viability after MPRO3 exposure was assessed using the MTT colorimetric assay. Cells were plated in 24-well culture plates and treated with MPRO3 at multiplicities of infection (MOIs) ranging from 1 to 200, or with vehicle alone, for a maximum of 3 days. At designated time points, the MTT reagent (5 mg/mL in PBS) was added to each well and incubated for 3 h. The resulting formazan product was dissolved in dimethyl sulfoxide, and its optical density was quantified at 570 nm using a microplate reader.

## Immunohistochemistry

Frozen tissue sections (5 μm) were incubated in PBS containing 0.3% $H_2O_2$, followed by blocking with 1% bovine serum albumin (BSA). The sections were then incubated with the primary antibodies. After washing, the sections were incubated with the EnVision Detection System (DAKO), counterstained with hematoxylin, and mounted using Vectamount (Vector Laboratories). Stained sections were imaged using a ZEISS Axio Scan.Z1 slide scanner (ZEISS).

## Supplementary Figures

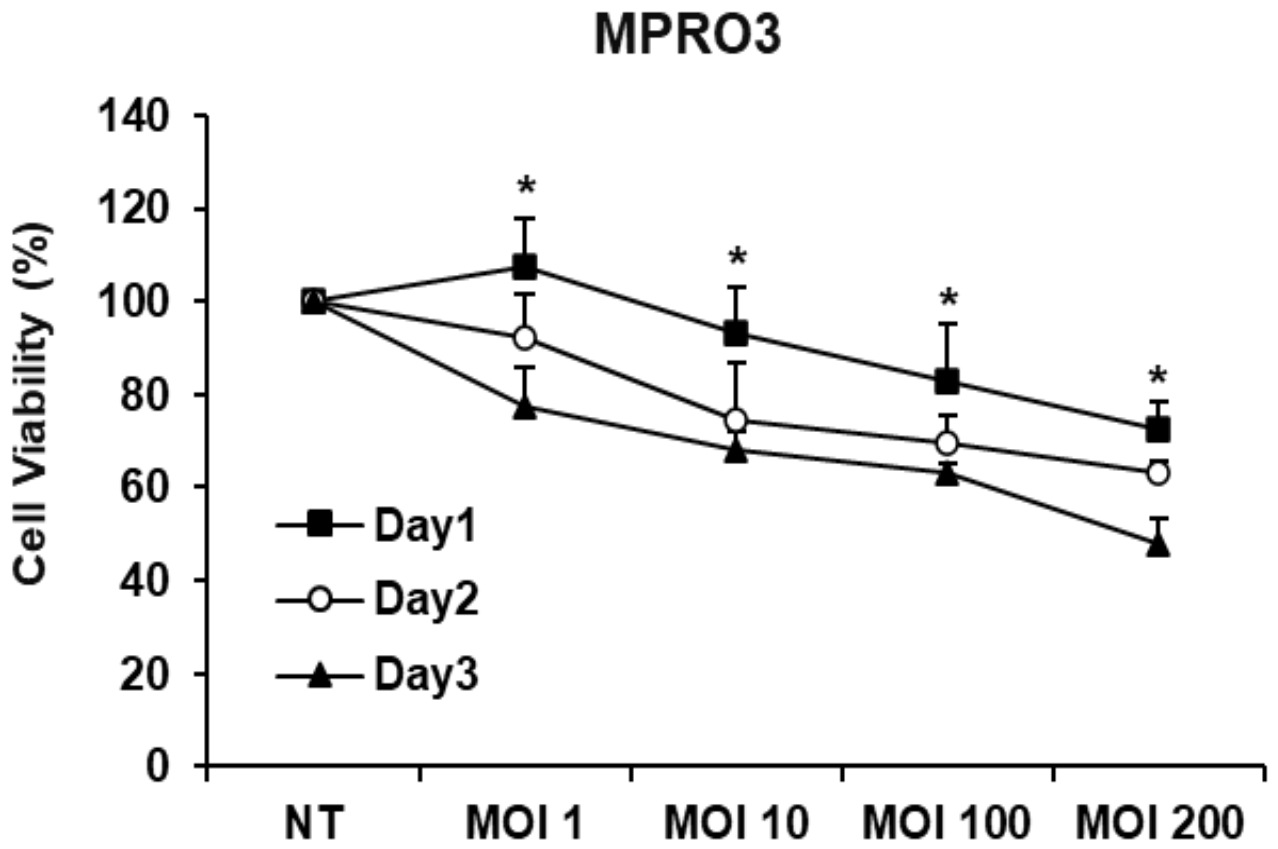


**Fig. S1. Effect of MPRO3 on the viability of THP-1 macrophages.** THP-1 monocytes were differentiated into resting macrophages (M0) by treatment with 150 nM PMA for 24 h and subsequently exposed to MPRO3 at the indicated MOIs. Cell viability was monitored daily for up to 3 days using an MTT assay.

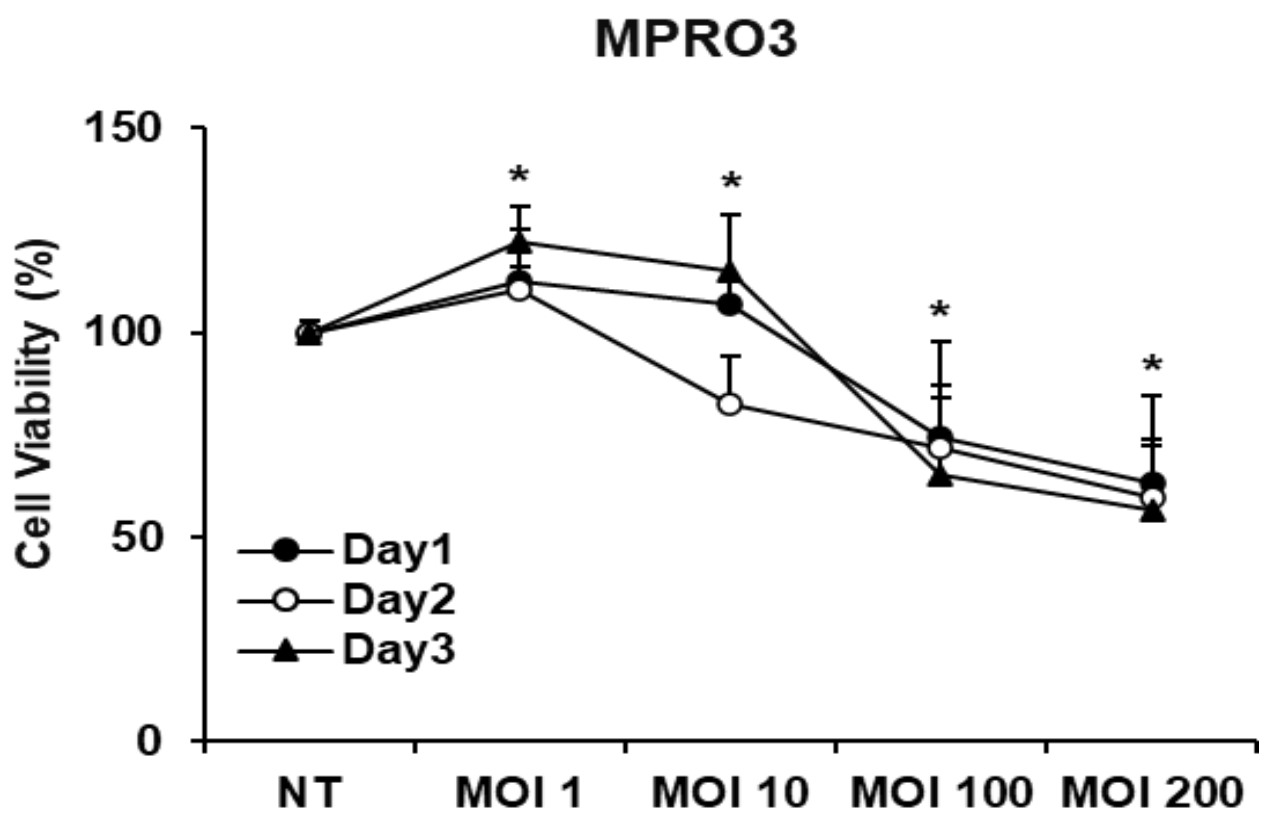


**Fig. S2. Effect of MPRO3 on the viability of BMMs.** BMMs were exposed to indicated MOIs of MPRO3 for 3 days and cell viability was determined daily by MTT assay.

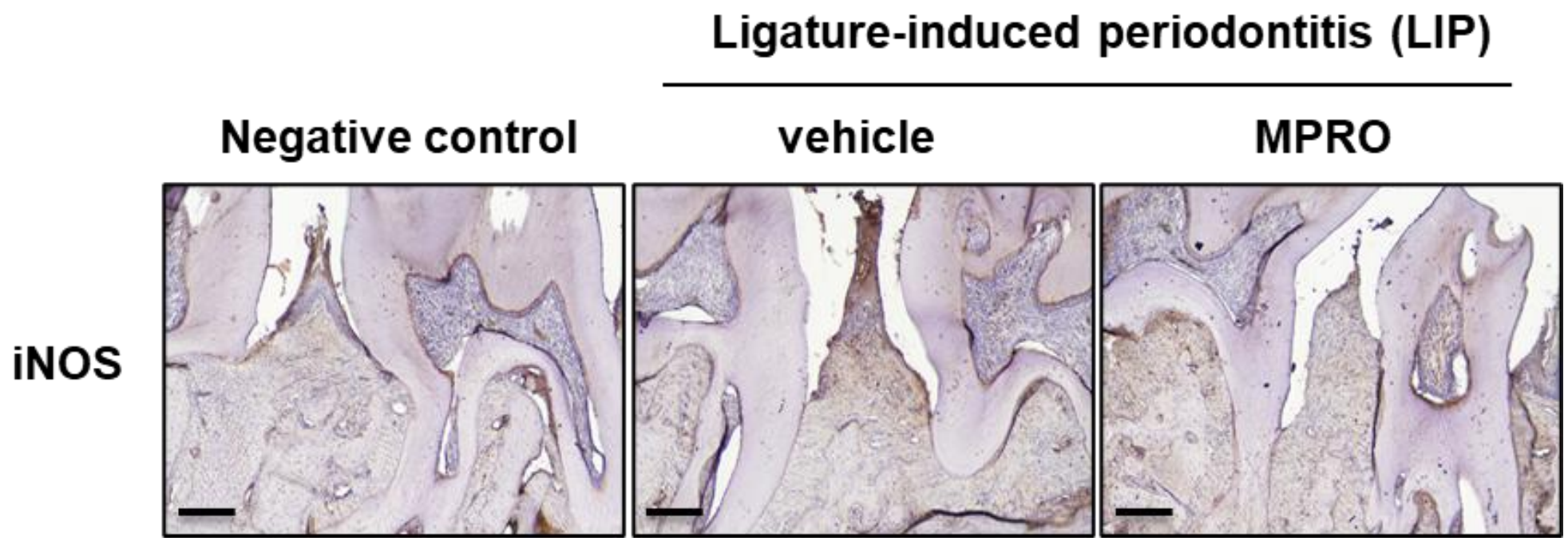


**Fig. S3. Effect of MPRO3 on M1 macrophage infiltration in gingival tissues of ligature-induced periodontitis mice.** Ligatures were placed around the left maxillary second molars, followed by daily oral administration of MPRO3 ($1 \times 10^9$ cells) for 14 days. Maxillary samples were collected at the end of the experimental period. Representative immunohistochemical staining of iNOS-positive M1 macrophages in gingival tissues is shown. Black bar = 100 μm.
